# Editorial: Imaging techniques to predict outcomes in renal cancers

**DOI:** 10.3389/fonc.2023.1349703

**Published:** 2024-01-04

**Authors:** Abhishek Mahajan, Shreya Shukla

**Affiliations:** ^1^ Department of Radiology, The Clatterbridge Cancer Centre NHS Foundation Trust, Liverpool, United Kingdom; ^2^ Department of Radiology, HBCH and MPMMCC, Tata Memorial Hospital, Varanasi, India

**Keywords:** editorial, RCC (Renal Cell Carcinoma), renal cancer, MRI, computed tomography

## Introduction

In the year 2020, GLOBOCAN data reported a global total of 431,288 new instances of kidney cancer, establishing it as the 14th most prevalent cancer on a worldwide scale. It ranks as the ninth most prevalent cancer in men and the 14th most common among women. Renal cell carcinoma (RCC) makes up the majority (90%) of these cases, with clear cell RCC at 70%, papillary RCC at 10–15%, and chromophobe RCC at 5%. Other subtypes are rare, constituting ≤1% of cases (1). The increased incidence of RCCs can be attributed in part to the rise in cross-sectional imaging in recent decades, leading to more incidentally detected renal masses. Despite rising incidence, kidney cancer has seen a mortality decline of about 2% per year from 2016 to 2020, underlining the impact of improved treatment (2).

Imaging plays a crucial role in the diagnosis and staging of RCC, guiding treatment, and posttreatment evaluation. Computed Tomography (CT) or Magnetic Resonance Imaging (MRI) are ideal imaging tools for the characterization of indeterminate renal lesions. The American Joint Commission on Cancer (AJCC) TNM system is most commonly used system for staging kidney cancer. Clinical staging, which includes physical examination, biopsy, and imaging findings, can pre-surgically determine the T stage, abnormal lymph nodes, venous invasion, and distant metastases. Imaging findings guide management decisions, including intervention choice and surgical approach (3).

Minimally invasive image-guided interventional radiology procedures are applicable in both localized and advanced diseases. Renal mass biopsy is indicated when a kidney lesion is suspected to be hematologic or metastatic. Percutaneous ablation is a potentially curative treatment option for T1a tumors (4). Clear cell RCC’s hypervascular nature makes Transarterial Embolization (TAE) a viable adjunctive technique or stand-alone therapy.

The 5-year survival rate among kidney cancer patients has steadily increased due to early tumor detection and more effective systemic therapy for advanced disease. However, one-third of patients are diagnosed with regional or distant metastases, and approximately one-fourth of those treated surgically with curative intent experience relapse with distant metastases (5). Targeted agents and immunotherapy have improved overall survival and prognosis, but responses vary among patients. Imaging biomarkers and response evaluation criteria have been developed to predict their efficacy.

This Research Topic, led by Dr. Mahajan and team, explores novel imaging techniques for predicting outcomes in renal cancers. The Research Topic includes nine manuscripts, comprising seven original research articles, one meta-analysis, and one review article. The following is a summary of each manuscript:

Diffusion Weighted Imaging (DWI) based diffusion kurtosis imaging (DKI) and Intravoxel incoherent motion (IVIM) models have evolved over recent years. In their original research, Cheng et al. studied the ability of DKI and IVIM to differentiate histologic grades and clinical stages of clear cell RCC.

The field of modern imaging has taken a giant leap with radiomics, now widely utilized in the diagnosis, prognostication, and post-treatment follow-up in tumor imaging. Radiomics involves extracting quantitative features from radiographic images, analyzing the heterogeneity of tumors throughout their volume, and developing predictive models that link imaging features with genomic patterns and clinical outcomes. This Research Topic includes four manuscripts specifically focused on the application of radiomics in renal cell carcinoma (RCC).

Xing et al. have applied contrast-enhanced CT-based radiomics to predict overall survival (OS) in RCC patients. Yin et al. have used radiomics features based on the differential network feature selection (FS) method to determine the feasibility of predicting WHO/ISUP grade and progression-free survival (PFS) of clear cell RCC. There is considerable overlap in the imaging appearance of Renal angiomyolipoma without visible fat and clear cell RCC which poses a challenge to radiologists. In their radiomics-based study, Wang et al. have created a scoring system to distinguish the two using CT imaging by establishing a model using logistic regression and weighted to be a scoring system.

In their original research, Huang et al. established an integrative nomogram model using contrast-enhanced CT features and underlying genomics patterns to predict the overall survival (OS) of clear cell RCC patients.

Liu et al. have explored the feasibility of utilizing urine metabolites for early clinical diagnosis of RCC by demonstrating different urine metabolic profiles in RCC patients, healthy and benign controls.

Kode et al. retrospectively analysed standard uptake values (SUVs) in patients with and without renal failure, finding that renal failure patients do not require a protocol adjustment for 2-deoxy-2-[18F]fluoro-d-glucose Positron Emission Tomography/Computed Tomography (PET/CT).

Pan et al. conducted a meta-analysis exploring the value of contrast-enhanced ultrasound (CEUS) in the diagnosis of renal cancer.

Liu has reviewed the usefulness of 18F-fluorodeoxyglucose FDG PET/CT in RCC, emphasizing its efficacy in postoperative surveillance and restaging when conventional imaging is inconclusive.

In conclusion, the editors express gratitude to all the contributors for their valuable input in this Research Topic, which aims to inspire future and novel research approaches in the field of imaging in renal cancers.

## Author contributions

AM: Conceptualization, Supervision, Writing – original draft, Writing – review & editing. SS: Conceptualization, Writing – original draft, Writing – review & editing.
